# The Efficacy of Resiliency Training Programs: A Systematic Review and Meta-Analysis of Randomized Trials

**DOI:** 10.1371/journal.pone.0111420

**Published:** 2014-10-27

**Authors:** Aaron L. Leppin, Pavithra R. Bora, Jon C. Tilburt, Michael R. Gionfriddo, Claudia Zeballos-Palacios, Megan M. Dulohery, Amit Sood, Patricia J. Erwin, Juan Pablo Brito, Kasey R. Boehmer, Victor M. Montori

**Affiliations:** 1 Knowledge and Evaluation Research Unit, Mayo Clinic, Rochester, Minnesota, United States of America; 2 Department of Health Sciences Research, Mayo Clinic, Rochester, Minnesota, United States of America; 3 Integrative Medicine Program–Division of General Internal Medicine, Mayo Clinic, Rochester, Minnesota, United States of America; 4 Mayo Graduate School, Mayo Clinic, Rochester, Minnesota, United States of America; 5 Division of Pulmonary and Critical Care Medicine, Mayo Clinic, Rochester, Minnesota, United States of America; 6 Mayo Clinic Libraries, Mayo Clinic, Rochester, Minnesota, United States of America; 7 Division of Endocrinology, Metabolism, and Nutrition, Mayo Clinic, Rochester, Minnesota, United States of America; Technion - Israel Institute of Technology, Israel

## Abstract

**Importance:**

Poor mental health places a burden on individuals and populations. Resilient persons are able to adapt to life’s challenges and maintain high quality of life and function. Finding effective strategies to bolster resilience in individuals and populations is of interest to many stakeholders.

**Objectives:**

To synthesize the evidence for resiliency training programs in improving mental health and capacity in 1) diverse adult populations and 2) persons with chronic diseases.

**Data Sources:**

Electronic databases, clinical trial registries, and bibliographies. We also contacted study authors and field experts.

**Study Selection:**

Randomized trials assessing the efficacy of any program intended to enhance resilience in adults and published after 1990. No restrictions were made based on outcome measured or comparator used.

**Data Extraction and Synthesis:**

Reviewers worked independently and in duplicate to extract study characteristics and data. These were confirmed with authors. We conducted a random effects meta-analysis on available data and tested for interaction in planned subgroups.

**Main Outcomes:**

The standardized mean difference (SMD) effect of resiliency training programs on 1) resilience/hardiness, 2) quality of life/well-being, 3) self-efficacy/activation, 4) depression, 5) stress, and 6) anxiety.

**Results:**

We found 25 small trials at moderate to high risk of bias. Interventions varied in format and theoretical approach. Random effects meta-analysis showed a moderate effect of generalized stress-directed programs on enhancing resilience [pooled SMD 0.37 (95% CI 0.18, 0.57) p = .0002; I^2^ = 41%] within 3 months of follow up. Improvement in other outcomes was favorable to the interventions and reached statistical significance after removing two studies at high risk of bias. Trauma-induced stress-directed programs significantly improved stress [−0.53 (−1.04, −0.03) p = .03; I^2^ = 73%] and depression [−0.51 (−0.92, −0.10) p = .04; I^2^ = 61%].

**Conclusions:**

We found evidence warranting low confidence that resiliency training programs have a small to moderate effect at improving resilience and other mental health outcomes. Further study is needed to better define the resilience construct and to design interventions specific to it.

**Registration Number:**

PROSPERO #CRD42014007185

## Introduction

### Rationale

Resilience has been defined as the ability of individuals to absorb life’s challenges and to carry on and persevere in the face of adversity. [1] Overlapping extensively with the concept of hardiness, psychological resilience personifies and reflects characteristics of toughness, elasticity, and the ability to recover. Although the term has been used in many disciplines and applied to many contexts, a recent concept analysis defined resilience as the “process of effectively negotiating, adapting to, or managing significant sources of stress or trauma.”[2].

When conceptualized in this way (i.e. as a response to stress or trauma), it is practically helpful to briefly consider the position resilience holds within a relevant stress model, such as Lazarus’ Transactional Model of Stress and Coping. According to this model, [3] many of the events that comprise the experience of life (i.e. illness, loss, trauma, new jobs or demands) can be considered “stressors.” In the absence of the resources needed to cope with and manage these stressors, people experience their effects in the form of reduced mental–and to a lesser extent physical–health. According to Lazarus’ model, then, the value of personal resilience lies in its potential as an internal resource for mitigating the negative effects of stress and for maintaining mental health through adversity [4].

Indeed, poor mental health places major constraints on the well-being, productivity, and prosperity of individuals, communities, and nations. [5] As such, there is widespread interest in better understanding and applying the mechanism by which resilience is able to avoid these constraints and promote health. [6]–[9] The predictors and effects of resilience have been examined among those living with chronic illness, overcoming traumatic experiences, and prospering in stressful work environments. Overall, research suggests that resilience is a modifiable construct and not an inherent, immovable trait of individuals. To the extent this is true, the potential public health impact of identifying and translating a reliable and efficacious method of achieving resilience in people is great.

Resiliency can be thought of as the process of achieving resilience. Clinicians, researchers, patients, public health agencies, governments, and others are investing heavily in mechanisms aimed at facilitating resiliency. Key among these, “resiliency training programs” are a loosely defined group of interventions that systematically seek to enhance resilience in individuals or groups. To our knowledge, no single accepted theoretical framework or consensus statement exists to guide the development or application of these programs. Furthermore, despite international use and testing, there remains little clarity related to what is fundamentally required for a program to be considered resiliency training, let alone for it to be considered effective. Indeed, one could argue that, without more guidance and understanding, the field runs the risk of overtranslating and/or diffusing its efforts.

To better understand the efficacy of resiliency training programs and to provide information that can benefit decision makers in directing future study, we sought to conduct a systematic review and meta-analysis. Clinically, we were particularly interested in the role resiliency training might play in improving the lives and health of patients with chronic conditions.

### Objectives

Our primary objective was to synthesize the evidence of resiliency training programs in improving resilience, quality of life, and self-efficacy and in reducing depression, stress, and anxiety in adults. A secondary aim was to determine the efficacy of these programs in patients with chronic conditions.

## Methods

A published protocol [10] (PROSPERO registration number CRD42014007185) guided the conduct of this review, which we report in adherence to the Preferred Reporting Items for Systematic Reviews and Meta-analyses (PRISMA) Statement [11].

### Eligibility Criteria

Eligible studies were randomized controlled trials published in any language assessing the efficacy of any program designed to develop or enhance resilience (or a related construct, “hardiness”) in adults. Eligible studies had to describe an intention to impact resilience or hardiness in their rationale or design. No eligibility restrictions were made based on the type of comparator used, the length of follow-up, or the outcomes measured. Studies that only evaluated dissemination and/or implementation of resiliency training programs were ineligible.

### Information Sources

In conjunction with an experienced research librarian (PJE), we searched the following electronic databases from 1990 to January 14, 2014: PubMed, Scopus, EBSCO CINAHL, Ovid MEDLINE, Ovid EMBASE, Ovid Cochrane Library, Web of Science, and Ovid PsycINFO. The complete electronic search strategy is available in **Supplement S1**. We also searched clinical trial registries, contacted experts and study authors, and hand searched bibliographies.

### Study Selection

After receiving formal instruction and piloting a small sample, a team of 7 reviewers (ALL, PRB, MRG, KRB, MMD, JBP, CZP) worked in duplicate and independently to screen out clearly ineligible papers by reading titles and abstracts and using a web-based software (Distiller SR). To aid in the identification of ongoing studies, reviewers were instructed to include study protocols of potentially eligible trials during this phase. Any conflicts warranted retrieval of a full text copy of the article and inclusion into the second phase of screening. During this phase, two reviewers (ALL, PRB) independently examined full text versions of candidate papers to determine final eligibility (kappa = 0.78). Study protocols were excluded at this stage after extraction of relevant author contact information; all conflicts were resolved by consensus.

### Data Collection

After piloting a standardized data extraction form, two reviewers (ALL, PRB) worked independently and in duplicate to extract details about the included trials’ participants, interventions, controls, outcomes, and risks of bias. Specific data extracted included the trial author, year of publication, setting, study objective, and type (patients, students, workforce, other) and demographics (age, gender, race) of participants. We extracted descriptions of the format and theoretical basis of the intervention and comparator, particularly noting whether the comparator was a well-matched attention control vs. not. We extracted information on the number of participants approached, enrolled, randomized, and analyzed when this was available. We extracted post-intervention means and standard deviations for six, a priori determined patient-reported outcome domains at both short (longest follow up≤3 months) and long (longest follow up ≥6 months) durations of follow-up.

The outcomes collected were patient-reported measures within the domains of 1) resilience, hardiness, or ability to cope; 2) quality of life or well-being; 3) patient activation, self-efficacy, or confidence for disease management; 4) depression; 5) stress; and 6) anxiety. A consensus of the authors was used to determine whether outcomes measured were appropriate for inclusion within a given domain. Each outcome was assigned a rating of “appropriate,” “inappropriate,” or “questionable” (see Appendix D). Only a single outcome was accepted within each domain for a given trial; when multiple outcomes existed within a single domain, a hierarchy was used that prioritized validated and frequently reported measures. When not reported, we calculated standard deviations from confidence intervals and standard errors and, when necessary, we estimated sample sizes from reported degrees of freedom. We imputed standard deviations in three cases [12]–[14] by using reported standard deviations from other trials using the same measure. To remain conservative, we used the largest standard deviation for each measure that we could find, prioritizing studies in comparable populations [15]–[17].

After extracting data, we emailed a standardized, pre-populated spreadsheet to all study authors to 1) confirm the accuracy of our extraction, 2) ascertain any missing information and, 3) inquire about other potentially eligible trials. Authors were given 10 days to respond before a second email was sent. If no response was received after the second email, we conducted an internet search to identify an alternative email or method of contact; if fruitful, a final contact attempt was made before declaring the author unreachable.

### Intervention Categorization

**Figure 1:**
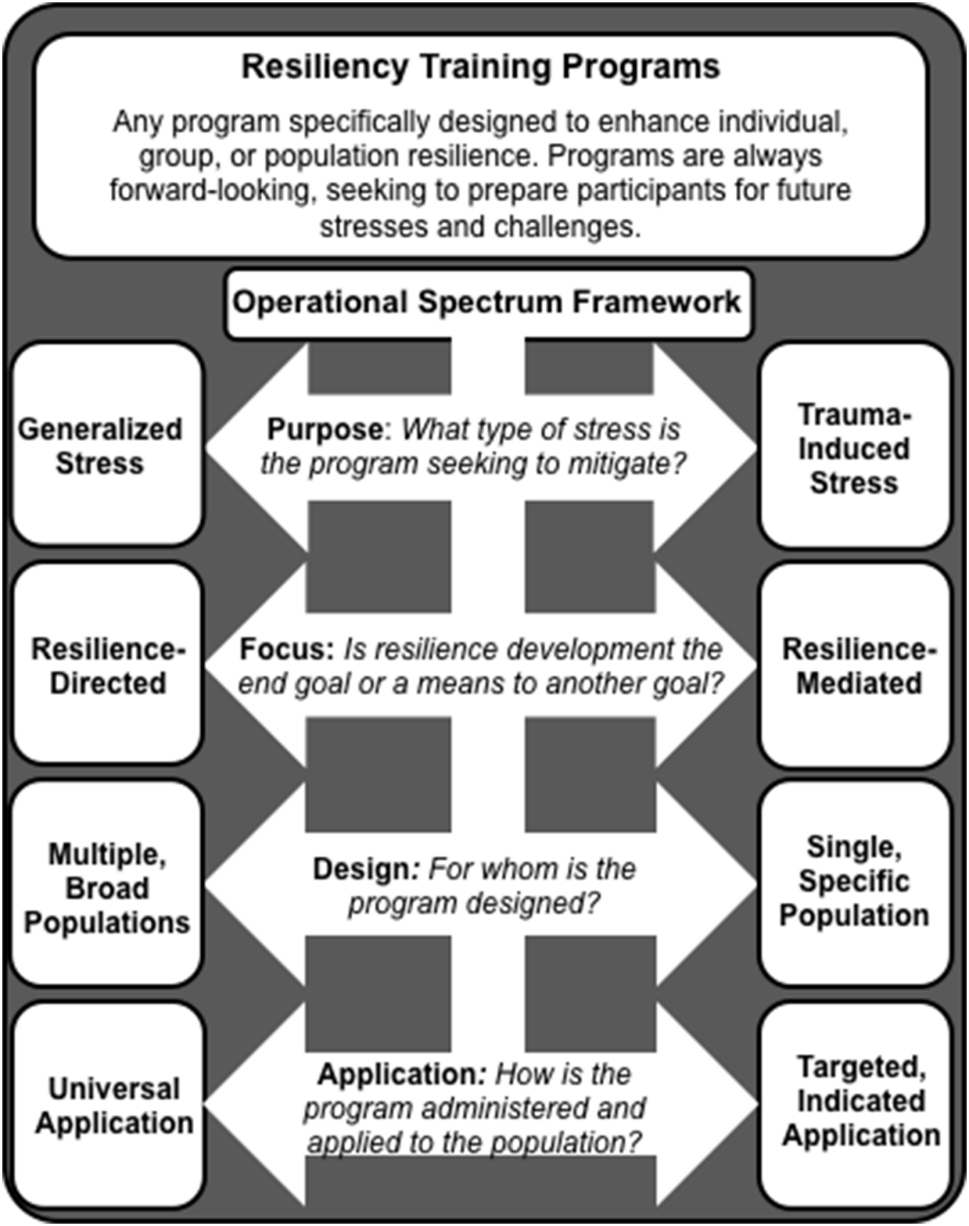
Resiliency training program operational framework.

Early in the review process, it became clear to us that study authors used diverse conceptual approaches when applying their training programs. For example, we found a particular dichotomizing distinction between programs based on the type of stress they sought to mitigate. Specifically, programs intending to impact trauma-induced stress (i.e. as might occur in individuals with post-traumatic stress disorder after a major catastrophe or tragic event) were very different in terms of approach used and outcomes evaluated from those intending to impact more generalized, every-day stresses. To aid in the organization, conceptualization, and analysis of the programs, we developed an ad hoc classification framework **(** **Figure 1**). This framework broadly classified training programs based on 1) whether they sought to mitigate generalized or trauma-induced stress, 2) whether they focused on developing resilience as an end goal or as a mediating variable, 3) whether they were designed to be used in single/specific or multiple/general populations, and 4) whether they were intended to be administered universally or in a targeted fashion (i.e. only “as needed”).

### Risk of Bias Within Studies

Risk of bias was assessed for each trial independently by two team members (AL, PB) using the Cochrane Collaboration’s Tool. [18] Specifically, we considered the quality of the randomization sequence generation; whether treatment arm allocation was concealed; the type and quality of blinding of participants, personnel, and outcome assessors; the degree and potential impact of missing data; the likelihood of incomplete reporting; and the potential role of conflicting interests. In cases where the intervention was explicitly intended to impact resilience and no measure of resilience was reported, we considered the study to be at high risk of selective reporting. We judged the potential impact of all biases on a given study’s reported outcomes and identified those studies at highest risk of bias. Particular weight was given to the impact of missing data, which was a well-distributed variable across studies. Conflicts in judgment were resolved through discussion and consensus.

### Data Synthesis

To permit pooling of effects across different measures of similar constructs, we converted the differences in post-intervention means to standardized mean differences (SMDs). Because of differences in the conceptual approaches of resiliency training programs designed to mitigate generalized stress compared to those specifically designed to impact post-traumatic stress–and in differences in the underlying psychobiology of these states–we elected, before looking at the data, to analyze these categories of programs separately. For both types of programs, when possible, we conducted a random effects meta-analysis of the SMDs within each of the six outcome domains collected. We assessed for between trial heterogeneity in excess of chance by calculating the I^2^ statistic. [19] We used RevMan Version 5.2 statistical software [20] for all analyses. Studies not reporting outcomes within the a priori domains or not reporting them at the level of the randomized participants (e.g. reporting changes in team or group culture as measured in different post-intervention samples) were not included in the meta-analyses.

### Risk of Bias Across Studies

Because included trials were small in size and few in number, it was inappropriate to assess for publication bias through planned funnel plot analyses. [21] Rather, we used global assessments of the body of evidence to postulate on its impact.

### Additional Analyses

We conducted planned subgroup analyses based on whether 1) the study participants had a chronic disease and 2) whether the trial had an attention control comparator. Because of heterogeneity in the format, structure, and theoretical approaches of programs, and the small number of trials for a given outcome, we were unable to formally assess the effects of intervention characteristics on outcomes.

We conducted sensitivity analyses based on the appropriateness of the included outcome (i.e. whether the outcome was rated as “questionable” for inclusion within a given domain), whether the study was judged at high risk of bias, and whether any required data was imputed.

## Results

### Study Selection

**Figure 2:**
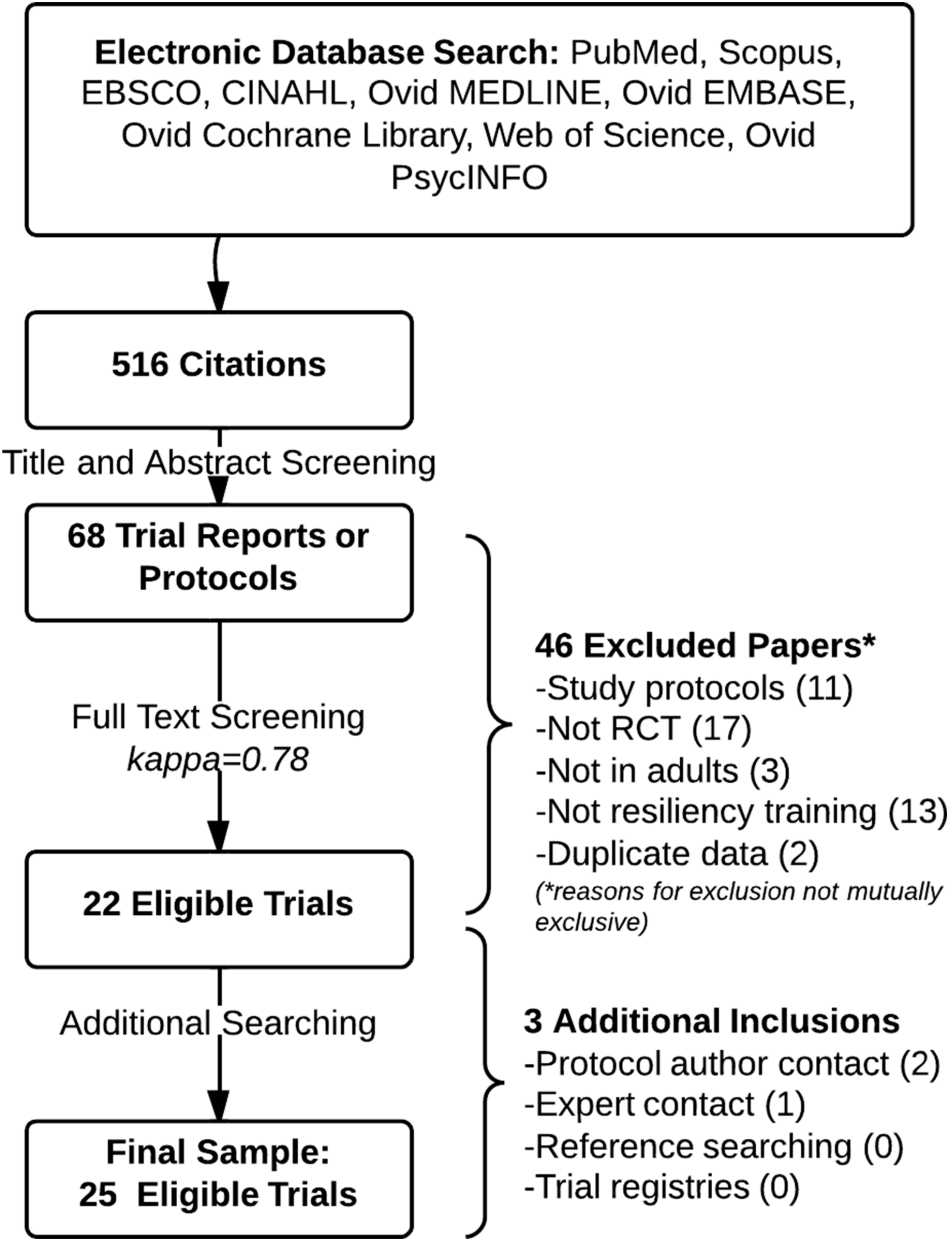
Study flow diagram.

The study flow diagram is presented in **Figure 2**. The electronic database search generated 516 candidate citations. Through title and abstract screening, we identified 68 potentially eligible trial reports or protocols. For these, we retrieved and reviewed full text versions, resulting in the inclusion of 22 trials. A complete list of full text papers reviewed and rationale for exclusion is provided in **Supplement S1**. Two additional trials were obtained through protocol author contact and one ongoing, eligible trial was identified through expert contact. Thus, the final sample consisted of 25 randomized trials ([13], [14], [22]–[42]; Sharma, unpublished data; and Burton, unpublished data). Authors responded to contact for 17 of the included studies but were often unable to provide additional data or information. A method of contact could not be identified for one study author [13].

### Study Characteristics

A summary of the included trials’ characteristics, including the theoretical basis and operational format of all interventions is presented in **Table 1**. In general, studies were small and conducted at single centers in diverse populations. Interventions varied widely in format, duration, and theoretical basis. Self-directed, electronic interventions; individual coaching or training sessions; and group courses and sessions were all tried with some efficacy across varying outcomes. Five studies evaluated programs designed to mitigate trauma-induced stress, while the remainder sought to impact stress more generally. Most trials were explicit in describing their intention to impact resilience, while three were less direct in describing this desire. [22], [33], [36] Two studies sought to impact resilience only as a mediator of a broader psychological construct. [30], [39] The theoretical bases of the tested interventions ranged from the use and application of well-established and/or resilience specific models and frameworks (i.e. The 5 C’s of Resilience, The Resilience Model, Lazarus’ Stress Model, etc.) to less clear and/or combined theoretical approaches drawing on broadly applicable strategies of stress management, attention interpretation, coping, and/or cognitive behavioral therapy. Most studies were of a wait-list control design, although 10 used an attention control.

**Table 1:** Summary of Included Study Characteristics and Findings.

Author, Year	Setting	Participants	Randomized; Analyzed	Intervention Description	Theoretical Basis	AttentionControlled	Framework Classification*	Summary of Findings and Follow-up	Outcomes Contributed toMeta-Analysis
**[22] Sahler,** **2013**	Multiple academic centers, USA	Mothers of children recently diagnosed with cancer; mean age 37; 43% white, 43% Hispanic	309; 191	8 weekly, 1-hr individual manualized, problem-solving skills training sessions	Five-step cognitive behavioral intervention designed to empower individuals through use of coping strategies and skills	Yes; non-directive support and reflective listening	Trauma, Directed, Specific, Universal	Continued, significant improvements in problem-solving skills, mood, anxiety, and post-traumatic stress at 3 months follow-up compared to non-directive support control	Depression, Stress
**[23] Rose, 2013**	Large University, USA	Volunteer, reimbursed graduate students with score of 16 or greater on Perceived Stress Scale; 50% male; 32% Asian	66; 59	6 weekly, 40-min, self-guided, computer-based, virtual resiliency training and homework/practice assignments with weekly calls/emails	Stress management training, cognitive behavioral approaches including thought activities and cognitive flexibility	Yes; stress-related videos and readings; with reminder emails/phone calls	General, Directed, Broad, Universal	Immediately post-test, improved amount of and control over stress in intervention group compared to educational attention control; intervention was rated as useful	Resilience, Stress
**[24] Varker, 2012**	Australian community	Compensated community volunteers; age 28; 56% female	82; 78	Single, 40 minute group session of stress inoculation training; included desensitization to car crash images and applied tension techniques	Stress inoculation training based on current understanding of PTSD	Yes; single session of pragmatic accident management training	Trauma, Directed, Specific, Universal	Analogue trial showed no significant difference in post-video distress but some improvement in affect at 1 month follow-up in MANOVA analysis	Depression, Stress
**[12], [25] Songprakun, 2012**	Outpatient psych hospital in Thailand	Moderately depressed Thai patients; age 42; 73% female	56; 53	8-week, resilience-focused self-help guide book with readings, homework, and weekly phone calls	Cognitive behavioral basis focusing on 4 resilience concepts to deal with depression	No; standard depression care only	General, Directed, Specific, Indicated	Significant improvement in resilience as add-on therapy in depressed Thai patients at 3 months follow-up	Resilience, Depression
**[26] Petree, 2012**	Restaurant franchises in Texas and Illinois	Young restaurant workers; age22; 52% female	28 restaurants and 485 workers randomized	3-day workshop, 2 hrs each day; focused on building team resilience	Focused on “five Cs” of resilience; modified for young adult perspective	No; no intervention	General, Directed, Specific, Universal	At 6 and 12 months follow-up, no significant difference in stress and problems with coworkers; outcomes were measured in different cohort than randomized	None
**[27] Sood, 2011**	Academic medical center	Department of Medicine faculty members; age 48; 47% female	40; 32	Individual, single, 90 minute session focused on attention training, deep breathing; optional follow-up session	Adapted from Attention and Interpretation Therapy; focuses on novelty of the world and living life with higher principles	No; wait list control	General, Directed, Broad, Universal	Significant improvement in resiliency, perceived stress, anxiety, and quality of life at 8 weeks of follow-up	Resilience, Quality of Life, Stress
**[28] Loprinzi, 2011**	Academic medical center, USA	Breast cancer survivors serving as peer mentors; age 61	24; 20	2, 90-minute small group sessions for attention training and relaxation, one optional 45 minute individual session and 3 follow-up calls over 12 wks	Adapted from Attention and Interpretation Therapy; focuses on novelty of the world and living life with higher principles	No; wait list control	General, Directed, Broad, Universal	Significant improvement in resilience, stress, anxiety, and quality of life at 3 months of follow-up	Resilience, Quality of Life, Stress
**[29] Kent, 2011**	VA Health System, USA	US Veterans with PTSD; age 54; 33%female	39; 39	12, manualized, weekly, 90-min group sessions to introduce concept of resilience, build self-awareness, test against stressors	Emphasizes a capacity-building approach to foster resilience resources that can be drawn upon	No; wait list control	Trauma, Directed, Broad, Indicated	Significant improvement in many affective symptoms and in emotional health immediately post-intervention	Depression, Stress
**[30] Luthans, 2010**	Large, Midwestern University, USA	Advanced management students; age 21; 42% female	NR; 153 in intervention and 89 in control	Single, 2-hr group training session with exercises and discussions to impact efficacy, hope, optimism, and resilience	Based on the construct of Psychological Capital (PsyCap) and developing its four loading capacity states	Yes; well-matched group decision-making intervention	General, Mediated, Broad, Universal	Significant and large improvement in Psychological Capital (PsyCap) at 3 days follow up in intervention group; res	None
**[33] Grant, 2010**	Independent girls school in Australia	High school educational workforce volunteers; age 43; 70% female	50; 44	10 individual coaching sessions over 20 weeks using GROW (goal, reality, options, way forward) model to structure conversations	Cognitive behavioral, solution-focused coaching based on self-leadership and role support plays in building resilience	No; wait list control	General, Directed, Broad, Universal	Increased goal attainment, reduced stress, and enhanced workplace well-being and resilience immediately post-intervention	Resilience, Depression, Stress, Anxiety
**[31] Farchi, 2010**	City in Israel during active war and bombing	Adult residents of Siderot, Israel; about 70% female	NR; 68	2 phone calls 1 wk apart that asked participants to refute 6 challenging sentences	Based on use of psychological inoculation to build resilience and coping efficacy	Yes; 2 phone calls asking about coping strategies used	Trauma, Directed, Broad, Universal	No significant difference at 1 wk of follow-up in mental resilience outcomes	None
**[32] Dolbier, 2010**	Large University, USA	Compensated student volunteers; age 21; 84% female; 25% Hispanic, 22% Asian	64; 38	4 weekly, 2 hr classroom sessions focused on transforming stress into resilience through empowering interpretations	Based on concept of stress-related growth, uses IFS model, CBT, transactional model of stress and coping and resilience models	No; wait list control	General, Directed, Broad, Universal	Evaluated reactions to remembered stressful events on stress-related growth; found no significant effect at 1 week of follow-up	None
**[37] Kanekar, 2009**	Midwestern University, USA	Asian-Indian volunteer students recently moving to US from India; age 25; 13% female	60; 39	3, self-directed online sessions to be completed over 2 months focusing on social support, hardiness, and acculturation	Based on previous research suggesting social support, hardiness, and acculturation predicted mental health in Indian students	Yes; similar format focusing on general health awareness and wellness	General, Directed, Specific, Universal	Immediate post-test improvement in psychological distress but no change in social support, hardiness, or acculturation	Resilience
**[36] Grant, 2009**	Public health nursing agency in Australia	Executives and senior managers in leadership development program; age 50; 93% female	50; 40	Individualized feedback, one half-day training workshop, then 4 individual sessions over 8–10 wks from executive coaches	Solution-focused, cognitive behavioral coaching using GROW model and applied positive psychology	No; control had the leadership workshop but no coaching sessions	General, Directed, Broad, Universal	Significant improvement in goal attainment, resilience, andworkplace well-being immediately post-intervention but not in anxiety or stress	Resilience, Depression, Stress, Anxiety
**[35] Arnetz, 2009**	Police academy in Sweden	Young, male, rookie Swedish police officers in parent study	25; 18	Initial psychoeducational session then 10 weekly, 2-hr group sessions for relaxation, imagery training, skill rehearsal; tapes for home practice	Stress inoculation training through imaginal exposure and visual motor behavioral rehearsal	No; typical police training	Trauma, Directed, Specific, Universal	After 1 year, improved psychophysiological stress and police work performance after robust, live critical incident simulation; no outcomes at shorter follow-up	None
**[34] Abbott, 2009**	Industrial organization in Australia	Sales manager volunteers; age 43; 14% female	53; 53	10 week, online, self-directed program using video, slides, virtual partners; also emails and a conference call	Focuses on emotion regulation, impulse control, optimism, causal analysis, empathy, self-efficacy, reaching out	No; wait list control	General, Directed, Broad, Universal	Half the sample was lost to follow-up but did ITT analysis; no significant difference immediately post-intervention between groups in mental health, QOL, or work performance; program well-accepted by completers	Quality of Life, Depression, Stress, Anxiety
**[38] Steinhardt, 2008**	Major University, USA	Compensated undergraduate and graduate students; age 21; 82% female; 26% Asian, 20% Hispanic	64; 57	4 weekly, 2-hr classroom sessions during final weeks of class; focus on transforming stress into resilience	Uses cognitive behavioral therapy drawing on transactional model of stress and coping and resilience and thriving models	No; wait list control	General, Directed, Broad, Universal	Significant improvement in resilience and coping immediately post-intervention and improved mental health symptomatology compared to control in MANOVA analysis	Resilience, Depression, Stress
**[39] Luthans, 2008**	Diverse industries through University contacts	Diverse sample of working adult volunteers; age 32; 89% white	NR; 364	2, 45-min, self-directed, web-based sessions 1 wk apart with narrated slides, reflection exercises, goal-setting	Based on Psychological Capital model and focuses on developing states of hope, optimism, efficacy, and resilience	Yes; attention-matched program focusing on decision making exercises	General, Mediated, Broad, Universal	Statistically significant but small improvement in PsyCap post-intervention compared to control	None
**[40] Bradshaw, 2007**	Academic diabetes center	Type 2 diabetes patients; age 59, 65% female; 90% white	200 randomized prior to invitation; 67 accepted; 51 analyzed	10, 90-min training classes 2x/wk for 5 wks held in the hospital	Modules focused on psychosocial enrichment, exploring functions to fortify mind, body, spirit	No; standard diabetes care	General, Directed, Specific, Universal	No significant improvement at 3 months in psychophysiological outcomes, although knowledge of coping strategies increased; outcomes not reported in fashion suitable for meta-analysis	None
**[41] Waite, 2004**	Government organization, USA	Members of work units in tax processing division; 84% female, 90% white	232 cluster randomized; 150 analyzed	5, weekly, 7-hr group sessions using Chi, quanta, practical experiences and skills	Focused on mental and spiritual health, development of understanding of disruptions	No; potential contamination with control work units	General, Directed, Broad, Universal	Significant improvement in resilience and psychological outcomes post-test; were maintained at 10 wks follow-up	Resilience, Self-efficacy
**[14] Schachman, 2004**	Air Force Base, USA	Primigravid military wives in childbirth class; age 21, 76% white	111; 91	Weekly group meetings x4 as part of traditional child birth course; small group and role play activities	Based on resilience model; sought to identify internal and external resources	Yes; attention-matched traditional classes	General, Directed, Specific, Universal	Significant and large improvement in resilience immediately post-intervention but not maintained 6 wks postpartum. Improved maternal role adaptation.	Resilience
**[13] Sadow, 1993**	Domiciliary for homeless veterans; USA	Substance-abusing homeless vets; likely all men	NR; 96	6 week program using metaphor of a scientist trying to make sense of life events	Psychoeducational instruction based on a resiliency training model, developing internal locus of control	Yes; program focused on verbal and written skills training	General, Directed, Broad, Universal	Significant but small improvement at 6 wks of follow-up in self-efficacy and internality of locus of control	Self-efficacy
**[42] Bekki, 2013**	Major Universities in USA	Compensated female doctoral students in physical sciences and engineering; age 27; 17% Asian	176; 134	Self-directed, web-based program explored for 5 hrs over 2 wks; uses case studies, video interviews	Problem-solving model; resilience and self-efficacy and cognitive behavioral theories	No; wait list control	General, Directed, Specific, Universal	Significant and large improvement immediately post-test in problem-solving knowledge, resilience, and coping efficacy compared to control	Resilience, Self-efficacy
**Sharma, unpublished**	Academic medical center, USA	Irritable bowel syndrome patients	23; 23 (interim analysis)	24 wk program with initial, individual training session, 6 handouts over next 12 wks; phone calls every 4 wks	Stress management and resilience training based on Attention Interpretation Training, mind-body approaches, transactional model of stress and coping	Somewhat; given stress management DVD and follow-up phone calls	General, Directed, Broad, Universal	Lost 3 to follow-up; interim ITT analysis half-way through intervention (12 wks) shows no significant difference in mental health or resilience outcomes	Resilience, Quality of Life, Stress, Anxiety
**[49] Burton, unpublished**	Single center, Australia	Worksite volunteers within a government department, clustered by occupation; age 45; 98% female	55 cluster randomized by occupation work site and geographic location; 46 analyzed	10 2.5 hr group sessions over 13 weeks targeting psychosocial well-being; involved psychoeducation, discussion, experiential learning, and home assignments with workbook; third arm not analyzed here added promotion of physical activity	Cognitive behavioral therapy based on six core Acceptance and Commitment Therapy processes	No, wait list control	General, Directed, Broad, Universal	At 1 month of follow up, no significant difference in most outcomes. A third arm not analyzed here involved the addition of physical activity to intervention and showed similar results. CHD risk indicators and physical activity also measured but not sought for this review.	Resilience, Depression, Stress, Anxiety

*Refers to operational framework presented in Figure 1. General = general stress-directed; Trauma = trauma-induced stress-directed; Directed = resilience-directed; Mediated = resilience-mediated; Broad = designed for broad/multiple populations; Specific = designed for specific/single populations; Universal = intended for universal application; Indicated = intended for “as needed” application

NR = not reported.

### Risk of Bias Within Studies

A summary of the risk of bias within each study is presented in **Supplement S1**. The risk of bias was judged to be moderate to high (agreement = 81%) for most studies. Unclear or incomplete reporting of methods and/or a high risk of missing data was frequently seen. In some cases, total numbers of subjects randomized and losses to follow-up were not reported and almost all studies conducted per protocol analyses. Seven studies were judged to have a particularly high risk of bias.([13], [26], [30]–[32], [34] and Burton, unpublished) We could not rule out a potential conflict of interest in six studies [26], [30], [33], [34], [36], [39].

### Results of Individual Studies

In general, resiliency training showed benefit in a number of mental health domains across diverse populations at ≤3 months of follow-up. In a number of cases, key variables needed for meta-analysis were not reported and could not be reliably imputed or obtained through author contact. To ensure the comprehensiveness of this review, we have summarized the results of all included studies in **Table 1**. For any given outcome, there was never more than one study reporting at a follow-up time ≥6 months. This precluded planned meta-analyses of the long-term effectiveness of resiliency training programs.

### Meta-analyses

**Figure 3:**
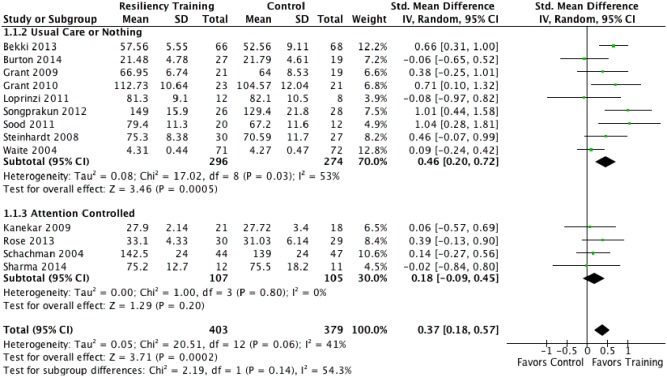
Forest plot of generalized stress-directed resiliency training programs’ effect (SMD) on resilience, divided into subgroups based on whether a well-matched attention control was used.

Across 13 contributing trials (782 participants), random effects meta-analysis showed an overall benefit of generalized stress-directed resiliency training in improving resilience in individuals within 3 months of follow-up [pooled SMD 0.37 (95% CI 0.18 to 0.57) p = .0002; I^2^ = 41%]. The estimated effect of these programs on quality of life and depression was also favorable but not statistically significant. Trauma-focused resiliency training programs showed a moderate effect in reducing stress symptoms [pooled SMD −0.53 (−1.04 to −0.03) p = .04; I^2^ = 73%] and a moderate effect in reducing depression [pooled SMD −0.51 (−0.92 to −0.10) p = .02; I^2^ = 61%]. A variety of measures were used within each of the outcome domains extracted. **Supplement S1** details the measures used and our rationale for including them in the pooled estimates of effect. A forest plot of the effects of resiliency training programs on resilience, divided into subgroups based on the presence of a well-matched attention control is presented in **Figure 3**. Forest plots for all other analyses can be found in **Supplement S1**. The complete results of the a priori meta-analyses, summarized by effect size, are presented in **Table 2**.

**Table 2:** Summarized Effects of Resiliency Training in Meta-analysis.

Outcome	No of Studies	Pooled Std. Mean Diff,Random Effects (95% CI)	P value	I^2^	Interpretation	Confidence∧
*Generalized stress-directed training programs*						
Resilience	13	0.37 (0.18, 0.57)	0.0002	41%	Small to moderate improvement	Moderate
Quality of life	4	0.34 (−0.03, 0.72)	0.07	10%	Non-significant improvement	Low
Self-efficacy	3	0.26 (−0.10, 0.63)	0.16	66%	Non-significant Improvement	Low
Depression	6	−0.28 (−0.56, 0.01)	0.06	33%	Non-significant improvement	Low
Stress	9	−0.28 (−0.60, 0.04)	0.09	57%	Non-significant improvement	Low
Anxiety	5	−0.11 (−0.41, 0.20)	0.48	17%	Non-significant improvement	Low
*Trauma-induced stress-directed training programs* *						
Depression	3	−0.51 (−0.92, −0.10)	0.02	61%	Moderate improvement	Low
Stress	3	−0.53 (−1.04, −0.03)	0.04	73%	Moderate improvement	Low
Anxiety	2	−0.61 (−1.54, 0.31)	0.19	81%	Non-significant improvement	Very low

*There were insufficient studies reporting resilience, quality of life, or self-efficacy outcomes to conduct meta-analysis.

***∧Based on a global assessment of risk of bias, appropriateness of measures, consistency of results, quality of controls, effect magnitude, and directness of the intervention among studies contributing to the outcome; possible ratings were “Very low,” “Low,” “Moderate,” and “High.”.***

### Risk of Bias Across Studies

The potential for publication and reporting bias was judged to be high. Of the 22 studies explicitly describing a desire to impact personal resilience, 10 failed to report an outcome measuring this construct. This was characteristic of trauma-directed [24], [29], [31], [35] and resilience-mediated [30], [39] training programs, which may have been less focused on resilience as a primary outcome. One study explicitly described a resilience-directed intervention and reported a resilience outcome in one paper, [25] but described the intervention’s purpose differently and reported different outcomes in other papers that were not captured by our initial database search. [12], [43] Of the 6 studies judged to have a potential conflict of interest, 4 failed to report a resilience outcome. Although the overall risk of bias for included studies was judged to be high, it was somewhat lower among the 18 studies contributing to the meta-analyses.

### Subgroup analyses

Among generalized stress-directed resiliency training programs, planned subgroup analyses based on whether an attention control was used or whether participants had a chronic disease failed to show a significant difference in intervention effect. Among studies evaluating trauma-directed resiliency training programs, both the non-attention-controlled and chronic disease subgroups comprised a single study conducted in patients with post-traumatic stress disorder (PTSD). [29] This study was significantly more effective at reducing depression (interaction p = .03), stress (interaction p<.01) and anxiety (interaction p = .02) than the other trauma-directed resiliency training programs. When a subgroup consists of a single study, however, observed effects are difficult to interpret and of limited value.

### Sensitivity Analyses

Sensitivity analyses based on whether an included outcome was rated as “questionable” for pooling appropriateness did not change interpretations. Of the seven studies judged to be at the highest risk of bias, three ([13], [34] and Burton, unpublished) contributed at least one outcome to the meta-analyses. Removal of the study by Sadow [13] did not change interpretation of the self-efficacy outcome. Removal of the studies by Abbott [34] and Burton (unpublished) however, independently resulted in increased estimates of the effect of resiliency training and reductions in heterogeneity across all included outcomes [resilience (Burton only), quality of life (Abbott only), and depression, stress, and anxiety (both Burton and Abbott)]. The study by Abbott lost about half of its sample to follow up and conducted an intention to treat (ITT) analysis; this likely underestimates the effectiveness of the intervention. The study by Burton used a cluster-randomized design that allocated participants by clusters according to type of employment and geographic location. The distribution of clusters was markedly unbalanced at baseline, however, and the treatment arms experienced different stressors at key points of data collection. Removing both of these studies from the analyses caused the estimated benefits in quality of life, depression, and stress to achieve statistical significance. The effects of their exclusion are summarized in **Table 3**.

**Table 3:** Effects of Removing Two Studies ([34] and Burton, unpublished) at High Risk of Bias from the Pooled Estimate of Generalized Stress-directed Training Program Effectiveness.

Outcome (number of studies)	Pooled Std. MeanDiff,Random Effects(95% CI)	P Value	I^2^	Absolute Change in Effect Size and New Interpretation
*Resilience*				
With Burton (13) 0.37(0.18, 0.57) 0.0002 41%	0.37 (0.18, 0.57)	0.0002	41%	+0.04; suggests a highly significant, moderately consistent, and moderate effect on improving resilience
Without Burton (12) 0.41 (0.20, 0.61) <0.0001 40%	0.41 (0.20, 0.61)	<0.0001	40%	
*Quality of Life*				
With Abbott (4)	0.34 (−0.03, 0.72)	0.07	10%	+0.28; suggests a significant, highly consistent, and moderate effect on improving quality of life
Without Abbott (3)	0.62 (0.14, 1.09)	0.01	0%	
*Depression*				
With Abbott/Burton (6)	−0.28 (−0.56, 0.01)	0.06	33%	−0.23; suggests a highly significant, highly consistent, and moderate effect on improving depression symptoms
Without Abbott/Burton (4)	−0.51 (−0.79, −0.22)	0.0005	0%	
*Stress*				
With Abbott/Burton (9)	−0.28 (−0.60, 0.04)	0.09	57%	−0.22; suggests a highly significant, highly consistent, and moderate effect on improving stress symptoms
Without Abbott/Burton (7)	−0.50 (−0.74, −0.26)	<0.0001	0%	
*Anxiety*				
With Abbott/Burton (5)	−0.11 (−0.41, 0.20)	0.48	17%	−0.26; suggests a borderline-significant, highly consistent, and small effect on improving anxiety symptoms
Without Abbott/Burton (3)	−0.37 (−0.75, 0.01)	0.06	0%	

## Discussion

### Summary of Findings

In general, the body of randomized trial evidence supports a modest but consistent benefit of resiliency training programs in improving a number of mental health outcomes within three months of follow-up. When excluding studies rated at high risk of bias, the estimated benefits are larger, more consistent, and more significant. Still, the overall methodological quality of included trials was low and several were poorly reported. We found no interaction with effect based on whether participants had chronic medical conditions. Although not statistically significant, we did identify a reduction in measured benefit in attention-controlled trials. Included studies were also small in number and size, which limits our ability to draw conclusions in high confidence.

There remains a lack of clarity related to what critically defines a resiliency training program. Programs are operationalized in diverse ways and lack a common theoretical or scientific specificity. The field also lacks a consistent approach to measurement [44] and it is often unclear whether outcomes chosen are sufficiently specific to the intervention. We developed a training program framework that helps to organize the operational approaches that have been taken in intervention design.

### Comparison With Prior Research

To our knowledge, this is the first systematic review and meta-analysis of resiliency training programs in adults, although a prior meta-analysis of a particular resiliency training program for children showed a similar effect in improving depression. [45] Our findings are also consistent with recent meta-analyses of meditation and mindfulness-based programs that showed efficacy in improving stress, depression, and well-being outcomes in clinical populations. [46]–[48] The effect sizes in these studies were comparable to those seen in our review, and may suggest similar value for resiliency training in patients with chronic conditions. Our subgroup analyses support this conclusion.

### Strengths and Limitations

We conducted this study according to a pre-defined and published protocol. To accumulate a high quality body of evidence, we restricted our inclusion to randomized trials and we searched databases and registries and contacted authors and experts to identify unpublished work. Still, this study has a number of limitations. First, our criteria for determining whether an intervention was a resiliency training program relied on our interpretations of the authors’ descriptions. We also combined a number of measures within construct domains. Despite efforts to account for the appropriateness of this approach, some uncertainty is inherent. The populations studied were heterogeneous and a normal distribution of outcomes was assumed in most cases; if this assumption were shown to be incorrect it would limit the validity of the pooled SMD estimates. Finally, we combined all outcomes reported within 3 months of follow-up. This approach gives a general impression of short-term program effectiveness but may overestimate the effect seen by excluding studies reporting outcomes immediately post-intervention.

### Implications

Clinicians, researchers, health policymakers, and governments are intrigued by the concept of resilience and the role it may play in promoting health and well-being. Finding reliable and effective ways to bolster resilience in individuals and populations is thus a key area of investigation. We have summarized the randomized trial evidence of programs designed to impact personal resilience.

### Future Study

To date, most studies related to resilience have been observational in nature. This may be an appropriate approach to further define the resilience construct and purposefully and scientifically design interventions to impact it. Research should focus on identifying a consistent and specific strategy for targeting resilience and a corresponding approach to measurement. When programs have clear scientific and theoretical rationale for effectiveness, they should be evaluated in larger, randomized controlled trials. In the future, comparative effectiveness studies will be needed to assess the specific and incremental value of resiliency training as compared to alternative programs (e.g. traditional cognitive behavioral therapy, mindfulness-based interventions, etc.). These trials should also have longer durations of follow-up to fully evaluate their effectiveness.

## Conclusions

Resiliency training programs seem to have benefit in improving mental health and well-being in diverse adult populations, although the quality of the randomized trial evidence precludes conclusions based in high confidence. There is no specific format, structure, or theoretical basis that defines a resiliency training program. In addition, no gold standard method of evaluation or measurement exists. Significant stakeholder interest in the potential of resiliency training programs warrants further study in this area. Such study should be rationally and scientifically organized, however, to achieve maximal value and fill key gaps in knowledge.

## Supporting Information

Checklist S1 PRISMA checklist for this review.
(DOC)

Data S1 Supplementary spreadsheet of all raw data used in analyses.
(XLSX)

Supplement S1 Supplementary file that includes the complete search strategy, a summary of excluded studies, the risk of bias assessments, a summary of pooled measures, and forest plots for all analyses.
(DOCX)
