# Supplementary material for: The Efficacy of Resiliency Training Programs: A Systematic Review and Meta-Analysis of Randomized Trials
Source: PLoS One. 2014 Oct 27;9(10):e111420. doi: 10.1371/journal.pone.0111420 (PMC4210242; doi:10.1371/journal.pone.0111420)

**The Efficacy of Resiliency Training Programs: A systematic review and meta-analysis of randomized trials**

**SUPPLEMENTAL MATERIALS**

**Appendix A:** Electronic search strategy page 2

**Appendix B:** Summary of excluded studies page 6

**Appendix C:** Risk of bias assessments page 13

**Appendix D:** Summary of pooled measures page 14

**Appendix E:** Forest plots of analyses page 22

**APPENDIX A: Electronic Search Strategy**

Resilience/resilient/hardiness

+

Building/enhance*

Training

intervention

Education/teach*

Increase*

Program

Promot*

Prevent*

**skills**

Scopus

TITLE-ABS-KEY(((resilient OR resiliency OR hardiness) AND (prevent* OR promot* OR enhanc* OR intervention* OR program* OR train* OR teach* OR educat* OR building OR increas*)) AND random*) AND SUBJAREA(mult OR agri OR bioc OR immu OR neur OR phar OR mult OR medi OR nurs OR vete OR dent OR heal OR mult OR arts OR busi OR deci OR econ OR psyc OR soci) AND (EXCLUDE(SUBJAREA, "AGRI") OR EXCLUDE(SUBJAREA, "BIOC") OR EXCLUDE(SUBJAREA, "ENVI") OR EXCLUDE(SUBJAREA, "COMP") OR EXCLUDE(SUBJAREA, "MATH"))  164

Scopus

(TITLE-ABS-KEY((resilien* OR hardiness) W/5 skills) AND NOT TITLE-ABS-KEY(child* OR pediatr* OR paediatri* OR adolescen*)) 122

PubMed

(resilient OR resiliency OR hardiness) AND (prevent* OR promot* OR enhanc* OR intervention* OR program* OR train* OR teach* OR educat* OR building OR increas*)) AND random*) NOT MEDLINE[sb] 14

| **Ovid MEDLINE(R)** 1946 to January Week 2 2014 | | | |
| --- | --- | --- | --- |
| **#** | **Searches** | **Results** | **Search Type** |
| 1 | Resilience, Psychological/ or resilience.mp. | 6163 | Advanced |
| 2 | 1 or resilient.mp. or resiliency.mp. or hardiness.mp. [mp=title, abstract, original title, name of substance word, subject heading word, keyword heading word, protocol supplementary concept word, rare disease supplementary concept word, unique identifier] | 9691 | Advanced |
| 3 | (prevent* or promot* or enhanc* or intervention* or program* or train* or teach* or educat* or building or increas*).mp. [mp=title, abstract, original title, name of substance word, subject heading word, keyword heading word, protocol supplementary concept word, rare disease supplementary concept word, unique identifier] | 5923234 | Advanced |
| 4 | 2 and 3 | 5391 | Advanced |
| 5 | limit 4 to (meta analysis or randomized controlled trial) | 159 | Advanced |
| 6 | limit 5 to (humans and yr="1990 - 2014") | 153 | Advanced |
| 7 | limit 6 to ("young adult (19 to 24 years)" or "adult (19 to 44 years)" or "young adult and adult (19-24 and 19-44)" or "middle age (45 to 64 years)" or "middle aged (45 plus years)" or "all aged (65 and over)" or "aged (80 and over)") | 98 | Advanced |
| 8 | 2 and (program evaluation/ or ed.fs. or patient education as topic/) | 276 | Advanced |
| 9 | limit 8 to (meta analysis or randomized controlled trial) | 19 | Advanced |
| 10 | limit 9 to (yr="1990 - 2014" and "all adult (19 plus years)") | 10 | Advanced |
| 11 | 7 or 10 | 98 |  |

| **EBM Reviews - Cochrane Central Register of Controlled Trials** December 2013 | | | |
| --- | --- | --- | --- |
| **#** | **Searches** | **Results** | **Search Type** |
| 1 | Resilience, Psychological/ or resilience.mp. | 165 | Advanced |
| 2 | 1 or resilient.mp. or resiliency.mp. or hardiness.mp. [mp=title, original title, abstract, mesh headings, heading words, keyword] | 264 | Advanced |
| 3 | (prevent* or promot* or enhanc* or intervention* or program* or train* or teach* or educat* or building or increas*).mp. [mp=title, original title, abstract, mesh headings, heading words, keyword] | 273807 | Advanced |
| 4 | 2 and 3 | 178 | Advanced |
| 5 | limit 4 to (meta analysis or randomized controlled trial) | 124 | Advanced |
| 6 | 2 and (program evaluation/ or ed.fs. or patient education as topic/) | 19 | Advanced |
| 7 | limit 6 to (meta analysis or randomized controlled trial) | 16 | Advanced |
| 8 | 5 or 7 | 124 | Advanced |
| 9 | limit 8 to yr="1990 - 2014" | 123 |  |

| **Embase** 1988 to 2014 Week 03 | | | |
| --- | --- | --- | --- |
| **#** | **Searches** | **Results** | **Search Type** |
| 1 | (resilient or resiliency or resilience or hardiness).mp. [mp=title, abstract, subject headings, heading word, drug trade name, original title, device manufacturer, drug manufacturer, device trade name, keyword] | 13139 | Advanced |
| 2 | exp training/ | 52815 | Advanced |
| 3 | 1 and 2 | 76 | Advanced |
| 4 | 1 and (program* or enhanc* or intervent* or train* or teach* or educat* or build* or increas*).mp. [mp=title, abstract, subject headings, heading word, drug trade name, original title, device manufacturer, drug manufacturer, device trade name, keyword] | 7286 | Advanced |
| 5 | (resilient or resiliency or resilience or hardiness).ti. and 4 | 1850 | Advanced |
| 6 | limit 5 to (embase and randomized controlled trial and yr="1990 - 2014") | 20 | Advanced |
| 7 | 3 or 6 | 94 |  |

Top of Form

| \| **PsycINFO** 1987 to January Week 2 2014 \| \| \| \| \| --- \| --- \| --- \| --- \| \| **#** \| **Searches** \| **Results** \| **Search Type** \| \| 1 \| exp "Resilience (Psychological)"/ and exp Training/ \| 99 \| Advanced \| \| 2 \| *"resilience (psychological)"/ \| 5174 \| Advanced \| \| 3 \| limit 2 to (adulthood <18+ years> and ("300 adulthood <age 18 yrs and older>" or 320 young adulthood <age 18 to 29 yrs> or 340 thirties <age 30 to 39 yrs> or 360 middle age <age 40 to 64 yrs> or "380 aged <age 65 yrs and older>" or "390 very old <age 85 yrs and older>") and yr="1990 - 2014") \| 2327 \| Advanced \| \| 4 \| (train* or program* or learn* or teach* or enhanc* or promot* or build* or increas* or educat*).mp. [mp=title, abstract, heading word, table of contents, key concepts, original title, tests & measures] \| 1189982 \| Advanced \| \| 5 \| 3 and 4 \| 1271 \| Advanced \| \| 6 \| 5 and random*.mp. [mp=title, abstract, heading word, table of contents, key concepts, original title, tests & measures] \| 68 \| Advanced \| |
| --- | --- | --- | --- | --- | --- | --- | --- | --- | --- | --- | --- | --- | --- | --- | --- | --- | --- | --- | --- | --- | --- | --- | --- | --- | --- | --- | --- | --- | --- | --- | --- | --- |

Added “skill*” to strategy, retrieved 22.

CINAHL

S7   S4 OR S5  Narrow by SubjectAge: - all adult Results (31)

S6  S4 OR S5   Results (104)

S5  S2 AND S3  Results (89)

S4 (MH "Hardiness/ED")  Limiters - Published Date: 19900101-20141231 Results (23)

S3 (MH "Social Skills Training") OR "training"  Limiters - Published Date: 19900101-20141231 Results (79,691)

S2  (MH "Hardiness")  Limiters - Published Date: 19900101-20141231    Results (2,821)

S1 (MH "Hardiness")  2,831

**APPENDIX B: Summary of Excluded Studies**

# Paper is a protocol or abstract:

| Refid | Title |
| --- | --- |
| Refid: 4Refid: 6Refid:122Refid:135Refid:158Refid:160Refid:221Refid:287Refid:316Refid:359Refid:363 | Evaluating the effectiveness of personal resilience and enrichment programme (PREP) for HIV prevention among female sex workers: a randomised controlled trial W. W. Y. Yuen, W. C. W. Wong, C. S. K. Tang, E. Holroyd, A. F. Y. Tiwari, D. Y. T. Fong, W. Y. Chin Level: 2, State: ExcludedNudging socially isolated people towards well-being with the 'Happiness Route': design of a randomized controlled trial for the evaluation of a happiness-based intervention L. A. Weiss, G. J. Westerhof, E. T. Bohlmeijer Level: 2, State: Excluded The TWIN-E project in emotional wellbeing: study protocol and preliminary heritability results across four MRI and DTI measures J. M. Gatt, M. S. Korgaonkar, P. R. Schofield, A. Harris, C. R. Clark, K. L. Oakley, K. Ram, H. Michaelson, S. Yap, M. Stanners, V. Wise, L. M. Williams Level: 2, State: Excluded  Resiliency training for mayo clinic residents and their patients R. P. Bright, M. Davis, D. F. Hurst, A. Zautra, T. B. Pipe, C. M. Stonnington, A. Mayer, J. M. Kling, Y. Ramos, L. E. Lough Level: 2, State: Excluded The applicability of 'Outcome Rating-Scale' and 'Session Rating-Scale' in an outpatient hospital setting S. Sourouri, L. Myklebust, I. Olsson Level: 2, State: Excluded  Cognitive training G. E. Smith Level: 2, State: Excluded  Destigmatizing mental illness C. Van Zelst, J. Van Os, Ph Delespaul Level: 2, State: Excluded  Promoting mental health in small-medium enterprises: an evaluation of the "Business in Mind" program A. Martin, K. Sanderson, J. Scott, P. Brough Level: 2, State: Excluded  Evaluating the effectiveness of psychosocial resilience training for heart health, and the added value of promoting physical activity: a cluster randomized trial of the READY program N. W. Burton, K. I. Pakenham, W. J. Brown Level: 2, State: Excluded  Work in progress - Using case studies to Increase the retention of female doctoral students in STEM Fields J. M. Bekki, B. L. Bernstein, K. Ellison, A. Sridharan, L. Hita, Q. Spadola Level: 2, State: Excluded  Fostering resilience in IT: A resource toolkit for faculty W. J. Smith, F. Bélanger, T. Lewis, K. Honaker Level: 2, State: Excluded |

# Is not a randomized control trial:

| Refid | Title |
| --- | --- |
| Refid: 26Refid: 38,Refid: 41Refid: 50Refid: 91Refid:148Refid:154Refid:155Refid:165Refid:201Refid:204Refid:237Refid:268Refid:333Refid:348Refid:349Refid:490 | The effect of integrating rational emotive behavior therapy and art therapy on self-esteem and resilience Mahmoud Roghanchi, Abdul Rashid Mohamad, See Ching Mey, Khoda Morad Momeni, Mohsen Golmohamadian Level: 2, State: Excluded  Evaluation of a standardized humor group in a clinical setting: a feasibility study for older patients with depression B. Konradt, R. D. Hirsch, M. F. Jonitz, K. Junglas Level: 2, State: Excluded  Patient training for psoriasis-evaluation of a standardized program H. Kling, J. Jahn, M. Sticherling Level: 2, State: Excluded  Physician resilience is an important skill S. Helmers, D. Van Winkle, K. Beine, A. Nedrow Level: 2, State: ExcludedDisseminating Self-Help: Positive Psychology Exercises in an Online Trial S. M. Schueller, A. C. Parks Level: 2, State: Excluded Does family intervention for adolescent substance use impact parental wellbeing? A longitudinal evaluation E. Yuen, J. W. Toumbourou Level: 2, State: Excluded  Supporting oncology health professionals: A review J. Turner, B. Kelly, A. Girgis Level: 2, State: Excluded  Factors predictive of resilience and vulnerability in new-onset epilepsy J. Taylor, A. Jacoby, G. A. Baker, A. G. Marson, A. Ring, M. Whitehead Level: 2, State: Excluded Enhanced response inhibition during intensive meditation training predicts improvements in self-reported adaptive socioemotional functioning B. K. Sahdra, K. A. MacLean, E. Ferrer, P. R. Shaver, E. L. Rosenberg, T. L. Jacobs, A. P. Zanesco, B. G. King, S. R. Aichele, D. A. Bridwell, G. R. Mangun, S. Lavy, B. A. Wallace, C. D. Saron Level: 2, State: Excluded The influence of short-term adventure-based experiences on levels of resilience A. Ewert, A. Yoshino Level: 2, State: Excluded Handling pressure : Developing skills for managers to stay resilient K. Cobb Level: 2, State: Excluded Computer-assisted resilience training to prepare healthcare workers for pandemic influenza: a randomized trial of the optimal dose of training R. G. Maunder, W. J. Lancee, R. Mae, L. Vincent, N. Peladeau, M. A. Beduz, J. J. Hunter, M. Leszcz Level: 2, State: Excluded  The impact of a newly designed resilience-enhancing programme on parent- and teacher-perceived resilience environment among Health Promoting Schools in Hong Kong M. C. Wong, J. Sun, A. Lee, D. Stewart, F. F. Cheng, W. Kan, M. Ho Level: 2, State: Excluded  A randomized group intervention trial to enhance mood and self-efficacy in people with multiple sclerosis S. A. Rigby, E. W. Thornton, C. A. Young Level: 2, State: Excluded  Long-term effects of bereavement and caregiver intervention on dementia caregiver depressive symptoms W. E. Haley, E. J. Bergman, D. L. Roth, T. McVie, J. E. Gaugler, M. S. Mittelman Level: 2, State: Excluded  Social ties and cognitive recovery after stroke: does social integration promote cognitive resilience? M. M. Glymour, J. Weuve, M. E. Fay, T. Glass, L. F. Berkman Level: 2, State: Excluded  Hardiness, stress, temperament, coping, and burnout in health professionals M. M. Rowe Level: 2, State: Excluded |

# Study is not conducted in adults:

| Refid | Title |
| --- | --- |
| Refid:317Refid:459Refid:472 | Exploring the feasibility of a therapeutic music video intervention in adolescents and young adults during stem-cell transplantation D. S. Burns, S. L. Robb, J. E. Haase Level: 2, State: Excluded  Reducing substance use and risky sexual behavior among young, low-income, Mexican-American women: comparison of two interventions C. S. Lindenberg, R. M. Solorzano, D. Bear, O. Strickland, C. Galvis, K. Pittman Level: 2, State: Excluded  Promoting resilience among children of sandwiched generation caregiving women through caregiver mutual help J. K. Tebes, J. T. Irish Level: 2, State: Exclude |

# Not felt to evaluate a resiliency training program:

#

| Refid | Title |
| --- | --- |
| Refid: 21Refid: 94Refid: 99Refid:136Refid:156Refid:171Refid:187Refid:188Refid:196Refid:208Refid:209Refid:394Refid:489 | Family bereavement program (FBP) approach to promoting resilience following the death of a parent I. N. Sandler, S. A. Wolchik, T. S. Ayers, J. Y. Tein, L. Luecken Level: 2, State: Excluded  Effects of positive psychology interventions in depressive patients-A randomized control study Reinhard Pietrowsky, Johannes Mikutta Level: 2, State: Excluded  Effect of cognitive behavioral therapy in mental health and hardiness of infertile women receiving assisted reproductive therapy (ART) L. Mosalanejad, A. K. Koolaee, S. Jamali Level: 2, State: Excluded  A comparison of cognitive bias modification for interpretation and computerized cognitive behavior therapy: effects on anxiety, depression, attentional control, and interpretive bias J. O. Bowler, B. Mackintosh, B. D. Dunn, A. Mathews, T. Dalgleish, L. Hoppitt Level: 2, State: Excluded  Functional assessment and positive support strategies for promoting resilience: Effects on teachers and high-risk children Karen Callan Stoiber, Maribeth Gettinger Level: 2, State: Excluded  Testing the effect of function-focused care in assisted living B. Resnick, E. Galik, A. Gruber-Baldini, S. Zimmerman Level: 2, State: Excluded  Building inner resilience in teachers and students Linda Lantieri, Eden Nagler Kyse, Susanne Harnett, Charlotte Malkmus Level: 2, State: Excluded  Competitive Memory Training (COMET) for low self-esteem in patients with personality disorders: a randomized effectiveness study K. Korrelboom, M. Marissen, T. van Assendelft Level: 2, State: Excluded  Mindfulness training increases momentary positive emotions and reward experience in adults vulnerable to depression: a randomized controlled trial N. Geschwind, F. Peeters, M. Drukker, J. van Os, M. Wichers Level: 2, State: Excluded  Using an experimental medicine model to explore combination effects of pharmacological and cognitive interventions for depression and anxiety M. Browning, M. Grol, V. Ly, G. M. Goodwin, E. A. Holmes, C. J. Harmer Level: 2, State: Excluded  Reducing heavy alcohol consumption in young restaurant workers K. M. Broome, J. B. Bennett Level: 2, State: Excluded  Insight and recovery from acute psychotic episodes M. Startup, M. C. Jackson, S. Startup Level: 2, State: Excluded   The efficacy and effectiveness of process consultation in improving staff morale and absenteeism R. Weir, L. Stewart, G. Browne, J. Roberts, A. Gafni, S. Easton, L. Seymour Level: 2, State: Excluded |

# Study presents duplicate data:

| Refid | Title |
| --- | --- |
| Refid: 86Refid:456 | Stress Management and Resilience Training (SMART) program to decrease stress and enhance resilience among breast cancer survivors: A randomized trial A. Sood, C. Loprinzi, V. Sharma, K. Prasad Level: 2, State: Excluded Determining the efficacy of resiliency training in the worksite Phillip John Waite Level: 2, State: Excluded |

**APPENDIX C: Risk of Bias Assessments**

**
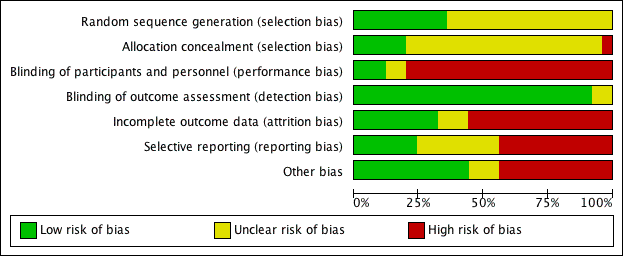
**


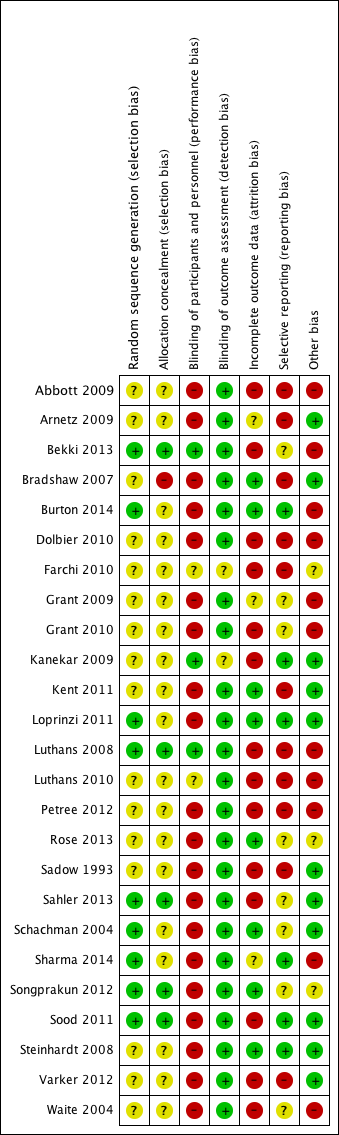


Studies Judged to Be at Highest Risk of Bias:

- Abbott, 2009
- Dolbier, 2010
- Petree, 2012
- Farchi, 2010
- Sadow, 1993
- Luthans, 2010
- Burton, 2014

Studies Judged to Be at Relatively Lower Risk of Bias

- Songprakun, 2012
- Sood, 2011
- Schachman, 2004
- Steinhardt, 2008
- Loprinzi, 2011

**APPENDIX D: Summary of Pooled Measures**

| Summary of Measures Pooled in Meta-analysis | | |  |
| --- | --- | --- | --- |
| Study | Measure Used | Description and Rationale | Domain Appropriateness (inappropriate measures not reported) |
| Resilience/Hardiness Measures | | |  |
| Rose, 2013 | Stress and Perception of Control Scale | Unpublished instrument developed for this study designed to assess perception of control and ability to cope with stress; Cronbach’s alpha was .71; attempts to contact author for further examination were unsuccessful | Questionable: sensitivity analysis did not show a change in interpretation |
| Songprakun, 2012 | 25-Item Resilience Scale; Thai translation | Well-studied instrument that was translated according to WHO process of translation and adaptation of instruments. Was pilot tested prior to study. | Appropriate |
| Sood, 2011 | Connor Davidson Resilience Scale | 25-item, widely-used scale that scored highly in recent review of resilience instruments | Appropriate |
| Loprinzi, 2011 | Connor Davidson Resilience Scale | 25-item, widely-used scale that scored highly in recent review of resilience instruments | Appropriate |
| Grant, 2010 | Cognitive Hardiness Scale | 18-item scale measuring control, commitment, and challenge (Kobasa); hardiness construct is measured as equivalent to resilience by several authors (i.e. dispositional resilience scale); the CHS has been used to measure resilience | Appropriate |
| Kanekar, 2009 | Hardiness Scale | 9-item version of CHS that has been used in Asian Indian population with acceptable reliability and validity | Appropriate |
| Grant, 2008 | Cognitive Hardiness Scale | 18-item scale measuring control, commitment, and challenge (Kobasa); hardiness construct is measured as equivalent to resilience by several authors (i.e. dispositional resilience scale); the CHS has been used to measure resilience | Appropriate |
| Steinhardt, 2008 | Connor Davidson Resilience Scale | 25-item, widely-used scale that scored highly in recent review of resilience instruments | Appropriate |
| Waite, 2004 | Modified Spirit Core Scale | Used 20 items and then modified to reflect the dimensions of innate resilience and the concept of reintegration; Cronbach’s alpha was 0.94; is an unpublished doctoral dissertation that focuses on spiritual aspects of resiliency; attempts to contact author for further examination were unsuccessful | Questionable: sensitivity analysis did not show a change in interpretation |
| Schachman, 2004 | Resilience Scale | Widely used in diverse populations; highly scored in recent review of resilience instruments | Appropriate, but standard deviations imputed for point of longest follow-up |
| Sharma, unpub | Connor Davidson Resilience Scale | 25-item, widely-used scale that scored highly in recent review of resilience instruments | Appropriate |
| Bekki, 1006 | A resilience scale developed for study | 16-item scale with Cronbach’s alpha of 0.77 and re-test reliability of 0.75; drew on Connor-Davidson and Wagnild’s Resilience Scale in development but modified to specifically fit population; validity unknown | Questionable: sensitivity analysis did not show a change in interpretation |
| Burton, 2014 | Brief Resilience Scale | 6-item instrument that has been found reliable; reasonably correlated with Connor Davidson | Appropriate |
| Overall Quality of Life/Well-Being Measures | | |  |
| Sood, 2011 | Single Item Overall Quality of Life Linear Analog Self Assessment Scale | Has been validated in the context of cancer patients to measure same construct as full measure; responsive to change | Appropriate |
| Loprinzi, 2011 | Overall Quality of Life Linear Analog Self Assessment Scale | Commonly used and well validated; responsive to change | Appropriate |
| Abbott | Authentic Happiness Inventory | Unpublished measure that is an updated measure of the Steen Happiness Index (which itself has good validity compared to other happiness measures). The AHI has had high internal consistency in university student samples. Chosen over WHOQOL-BREF because this measure was not reported as a composite or with details needed for meta-analysis. | Questionable: sensitivity analysis reduces heterogeneity and changes interpretation of effect such that it achieves statistical significance |
| Sharma, 1000 | Single Item Overall Quality of Life Linear Analog Self Assessment Scale | Has been validated in the context of cancer patients to measure same construct as full measure; responsive to change | Appropriate |
| Activation/Self-efficacy Measures | | |  |
| Waite, 2004 | Multidimensional Locus of Control Scale; internal subscale | 8 items that measure the degree to which one perceives events in life as being a consequence of ones own acts; this was felt to be consistent with the construct of self-efficacy, although Cronbach’s alpha ranged only from 0.64 to 0.71 | Appropriate |
| Sadow, 1993 | Self Efficacy Scale from Sherer (1982) | We could not obtain standard deviations to pool this outcome so it was not included. | Appropriate, but imputed standard deviations |
| Bekki, 2013 | Coping Efficacy | 14 item scale with Cronbach’s alpha of 0.91 that was specifically designed to measure self efficacy to overcome barriers that female STEM graduate students might encounter; measure would not generalize | Appropriate reflection of outcome in this specific population; elected to include in pooled estimate |
| Depression Measures | | |  |
| Songprakun, 2013 | Center for Epidemiologic Studies Depression Inventory | Commonly used and validated instrument assessing symptoms over the past week | Appropriate, but imputed standard deviations |
| Grant, 2010 | Depression Anxiety and Stress Scale-21; depression subscale | Commonly used and validated instrument assessing symptoms over the past week | Appropriate |
| Grant, 2009 | Depression Anxiety and Stress Scale-21; depression subscale | Commonly used and validated instrument assessing symptoms over the past week | Appropriate |
| Abbott, 2009 | Depression Anxiety and Stress Scale-21; Square Root Transformed; depression subscale | Commonly used and validated instrument assessing symptoms over the past week; square root transformation accounts for non-normalized data and can be meta-analyzed when converting to a standardized scale | Appropriate |
| Steinhardt, 2008 | Center for Epidemiologic Studies Depression Inventory | Commonly used and validated instrument assessing symptoms over the past week | Appropriate |
| Sahler, 2013 | Beck Depression Inventory II-Sq rt Transformed | Self-assessment of depressive symptoms over past two weeks; widely used with good reliability; square root transformation accounts for non-normalized data and can be meta-analyzed when converting to a standardized scale | Appropriate |
| Varker, 2012 | Depression Anxiety and Stress Scale-21; depression subscale | Commonly used and validated instrument assessing symptoms over the past week | Appropriate |
| Kent, 2011 | Beck Depression Inventory II | Self-assessment of depressive symptoms over the past two weeks; widely used with good reliability | Appropriate |
| Burton, 2014 | Depression Anxiety and Stress Scale-21; depression subscale | Commonly used and validated instrument assessing symptoms over the past week | Appropriate |
| Stress Measures | | |  |
| Rose, 2013 | Modified Perceived Stress Scale-10 | 10 items assessing perceived stress over the past month that was modified to ask about prior two weeks; commonly used | Appropriate |
| Sood, 2011 | Perceived Stress Scale | Widely used, 10-item, validated instrument assessing symptoms over the past month | Appropriate |
| Loprinzi, 2011 | Perceived Stress Scale | Widely used, 10-item, validated instrument assessing symptoms over the past month | Appropriate |
| Grant, 2010 | Depression Anxiety and Stress Scale-21; stress subscale | Commonly used and validated instrument for assessing symptoms over the past week | Appropriate |
| Grant, 2009 | Depression Anxiety and Stress Scale-21; stress subscale | Commonly used and validated instrument for assessing symptoms over the past week | Appropriate |
| Abbott, 2009 | Depression Anxiety and Stress Scale-21; stress subscale | Commonly used and validated instrument for assessing symptoms over the past week | Appropriate |
| Steinhardt, 2008 | Perceived Stress Scale | Widely used, 10-item, validated instrument assessing symptoms over the past month | Appropriate |
| Sharma, unpub | Perceived Stress Scale | Widely used, 10-item, validated instrument assessing symptoms over the past month | Appropriate |
| Sahler, 2013 | Impact of event Scale-Revised | Established reliability and validity for assessing post-traumatic stress to specific event | Appropriate for trauma |
| Varker, 2012 | Depression Anxiety and Stress Scale-21; stress subscale | Commonly used and validated instrument assessing symptoms over the past week | Appropriate |
| Kent, 2011 | Posttraumatic Stress Diagnostic Scale | Validated for detection and diagnosis of post-traumatic stress. Assesses symptoms over the last month. Would not generalize to non-traumatic stress. | Appropriate for trauma |
| Burton, 2014 | Depression Anxiety and Stress Scale-21; stress subscale | Commonly used and validated instrument assessing symptoms over the past week | Appropriate |
| Anxiety Measures | | |  |
| Grant, 2010 | Depression Anxiety and Stress Scale-21; anxiety subscale | Commonly used and validated instrument assessing symptoms over the past week | Appropriate |
| Grant, 2009 | Depression Anxiety and Stress Scale-21; anxiety subscale | Commonly used and validated instrument assessing symptoms over the past week | Appropriate |
| Abbott, 2009 | Depression Anxiety and Stress Scale-21; Square Root Transformed; anxiety subscale | Commonly used and validated instrument assessing symptoms over the past week; square root transformation accounts for non-normalized data and can be meta-analyzed when converting to a standardized scale | Appropriate |
| Sharma, unpub | Generalized Anxiety Disorder-7 | 7-item, validated instrument for assessing generalized anxiety symptoms over the past 2 wks | Appropriate |
| Varker, 2012 | Depression Anxiety and Stress Scale-21; anxiety subscale | Commonly used and validated instrument assessing symptoms over the past week | Appropriate |
| Kent, 2011 | State-Trait Anxiety Inventory-State | Designed to measure anxiety induced by perceived threat that is often considered temporary; would be less appropriate for generalized stress-directed interventions than trauma-induced ones. It assesses symptoms at that given time. | Appropriate for trauma |
| Burton, 2014 | Depression Anxiety and Stress Subscale-21; anxiety subscale | Commonly used and validated instrument assessing symptoms over the past week | Appropriate |

**APPENDIX E: Forest Plots of Meta-analyses**

**Generalized Stress-directed Training Programs:**

**Resilience:** attention-controlled subgroups


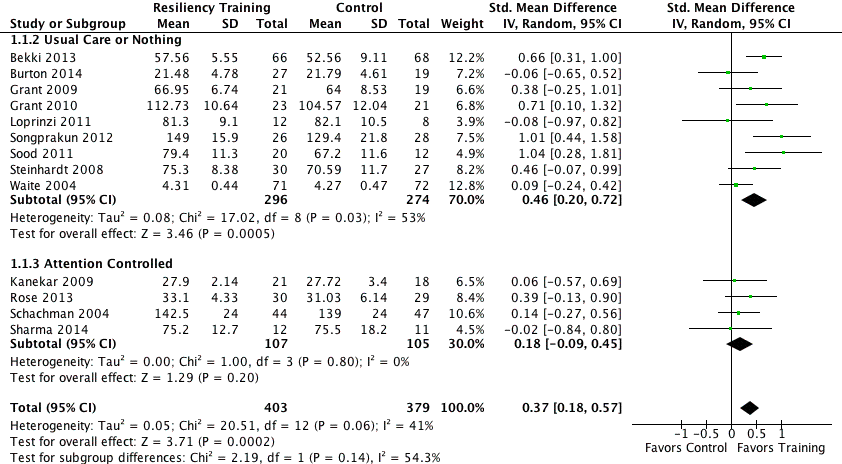


**Resilience:** chronic disease subgroups


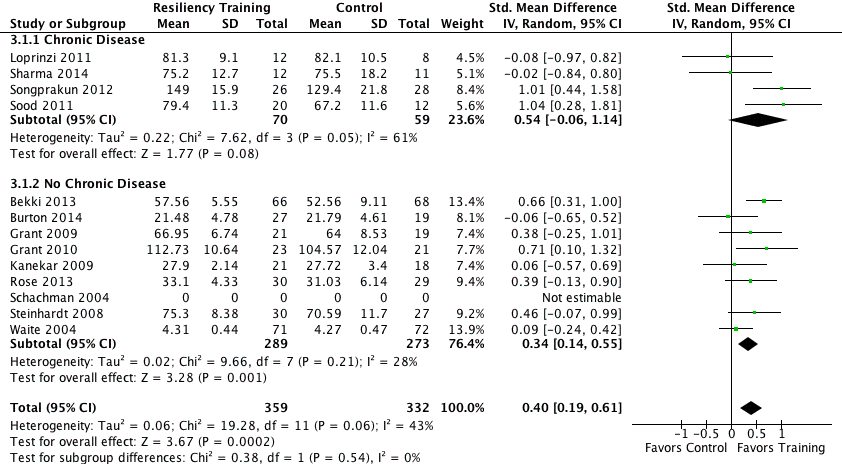


**Resilience:** risk of bias sensitivity analysis

**
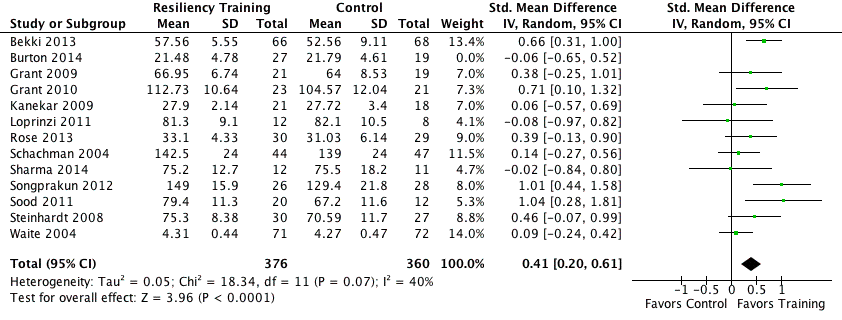
**

**Quality of Life:** attention-controlled subgroups


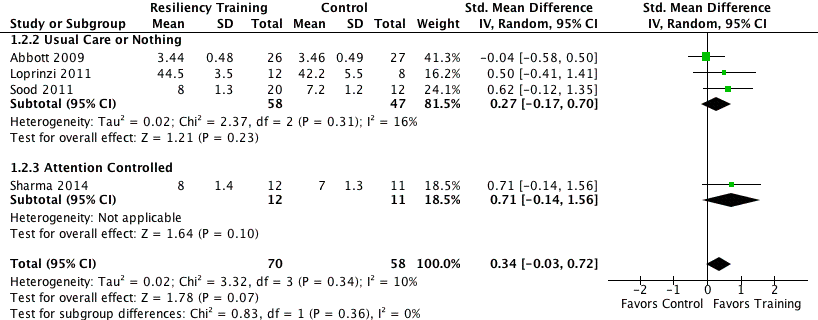


**Quality of Life:** chronic disease subgroups


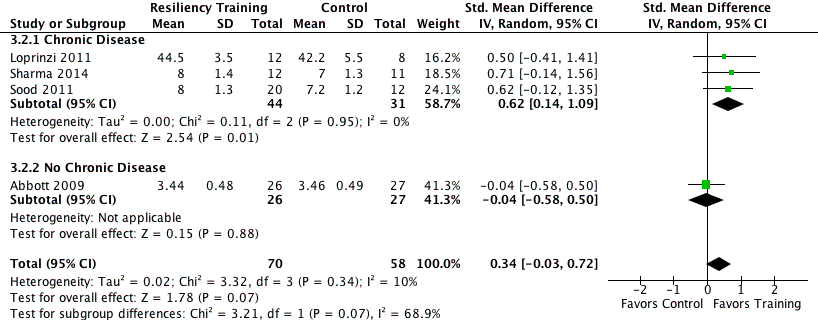


**Quality of Life:** risk of bias sensitivity analysis


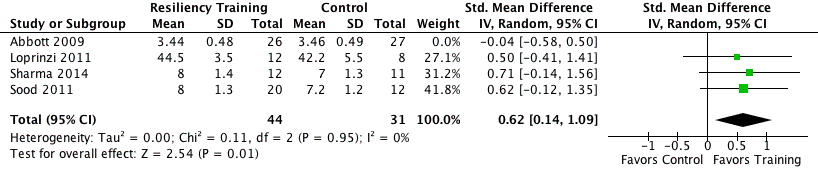


**Self-efficacy:** attention-controlled subgroups


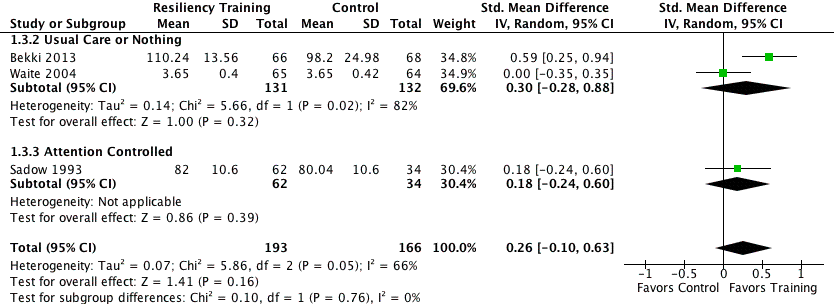


**Self-efficacy:** chronic disease subgroups


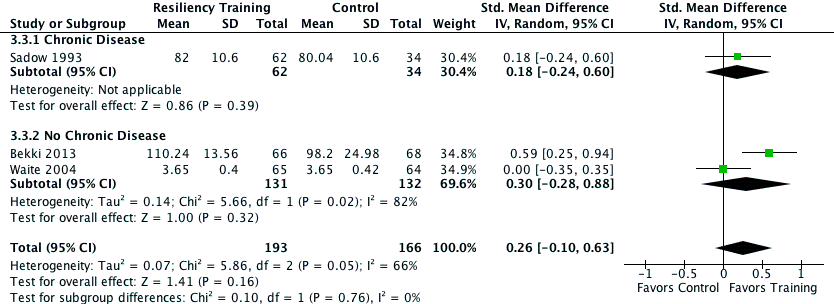


**Depression:** attention-controlled subgroups


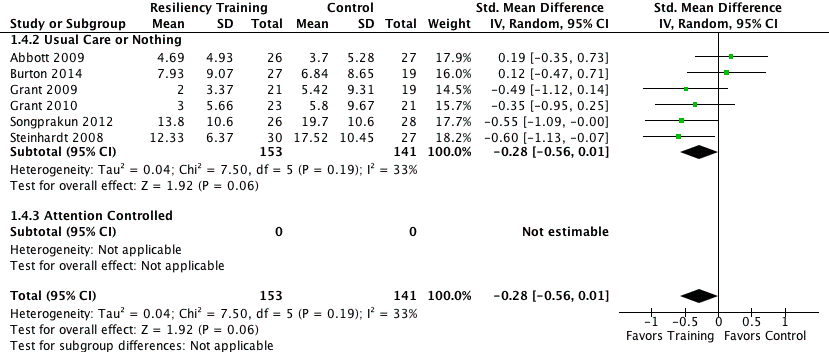


**Depression:** chronic disease subgroups


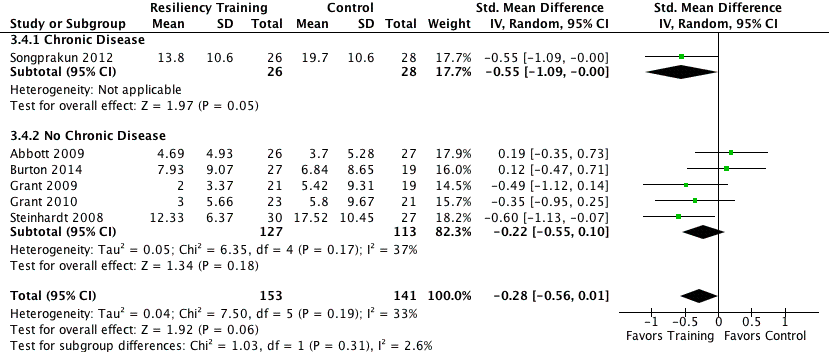


**Depression:** risk of bias sensitivity analysis


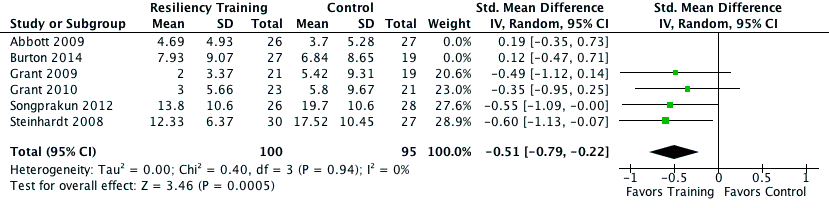


**Stress:** attention-controlled subgroups


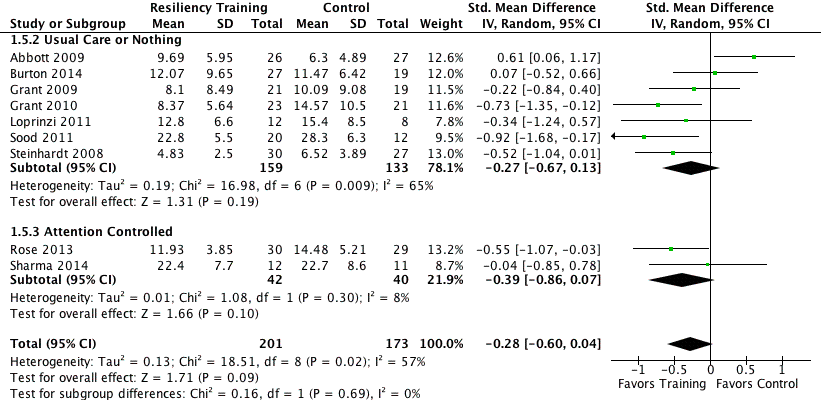


**Stress:** chronic disease subgroups


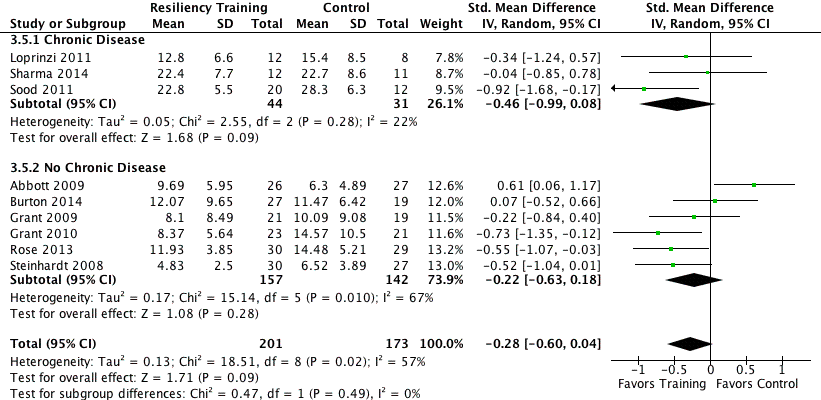


**Stress:** risk of bias sensitivity analysis

**
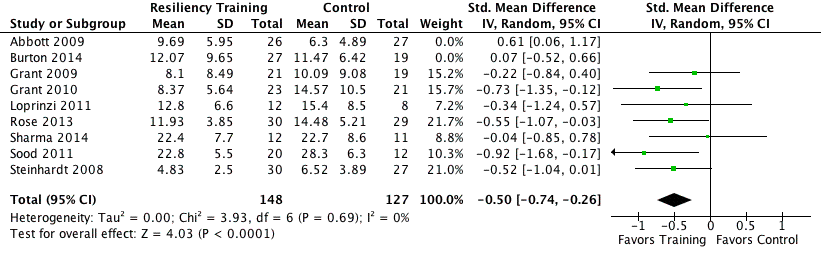
**

**Anxiety:** attention-controlled subgroups


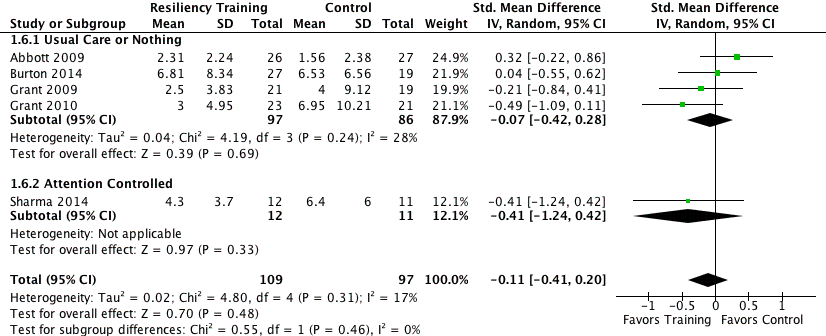


**Anxiety:** chronic disease subgroups


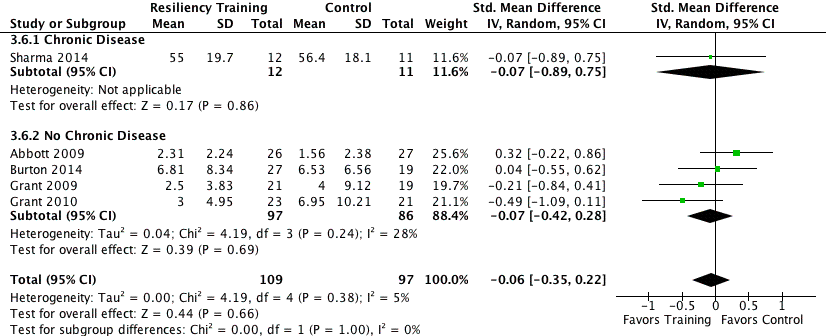


**Anxiety:** risk of bias sensitivity analysis


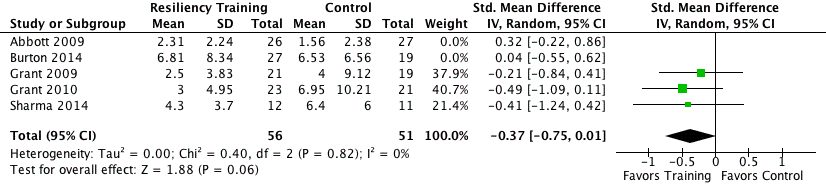


**Trauma-directed Training Programs:**

**Depression:** attention-controlled and chronic disease subgroups


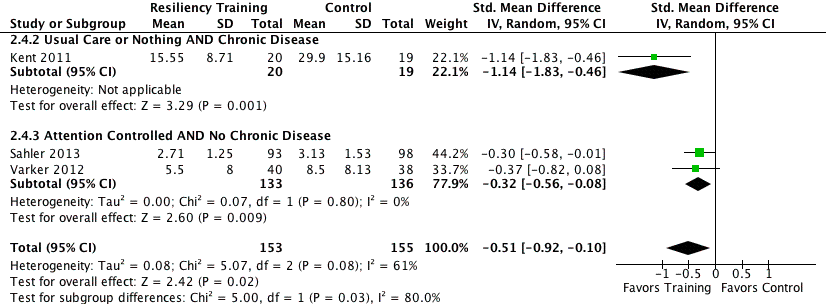


**Stress:** attention-controlled and chronic disease subgroups


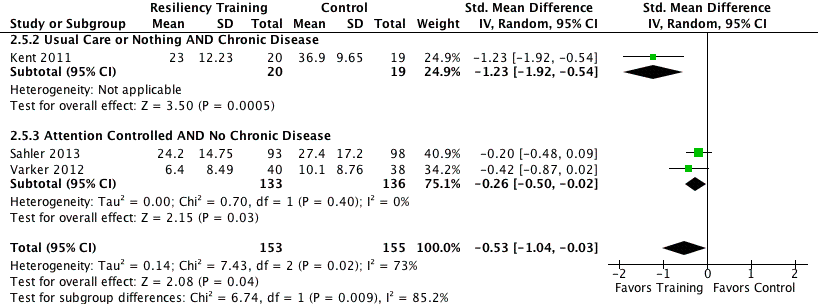


**Anxiety:** attention-controlled and chronic disease subgroups


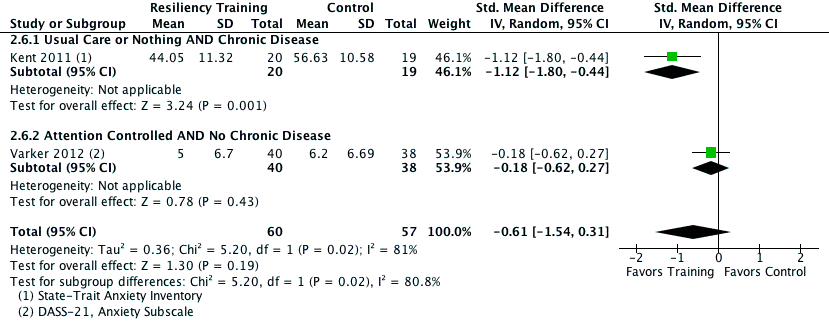

Supplement: Supplement S1 — Supplementary file that includes the complete search strategy, a summary of excluded studies, the risk of bias assessments, a summary of pooled measures, and forest plots for all analyses. (DOCX) [file pone.0111420.s003.docx]
